# α5β1 Integrin-Mediated Adhesion to Fibronectin Is Required for Axis Elongation and Somitogenesis in Mice

**DOI:** 10.1371/journal.pone.0022002

**Published:** 2011-07-22

**Authors:** Amparo Girós, Katja Grgur, Achim Gossler, Mercedes Costell

**Affiliations:** 1 Departament de Bioquimica i Biologia Molecular, Universitat de València, Burjassot, Spain; 2 Department of Molecular Medicine, Max Planck Institute of Biochemistry, Martinsried, Germany; 3 Institute for Molecular Biology, Medizinische Hochschule Hannover, Hannover, Germany; University of Bergen, Norway

## Abstract

The arginine-glycine-aspartate (RGD) motif in fibronectin (FN) represents the major binding site for α5β1 and αvβ3 integrins. Mice lacking a functional RGD motif in FN (FN^RGE/RGE^) or α5 integrin develop identical phenotypes characterized by embryonic lethality and a severely shortened posterior trunk with kinked neural tubes. Here we show that the FN^RGE/RGE^ embryos arrest both segmentation and axis elongation. The arrest is evident at about E9.0, corresponding to a stage when gastrulation ceases and the tail bud-derived presomitic mesoderm (PSM) induces α5 integrin expression and assumes axis elongation. At this stage cells of the posterior part of the PSM in wild type embryos are tightly coordinated, express somitic oscillator and cyclic genes required for segmentation, and form a tapered tail bud that extends caudally. In contrast, the posterior PSM cells in FN^RGE/RGE^ embryos lost their tight associations, formed a blunt tail bud unable to extend the body axis, failed to induce the synchronised expression of *Notch1* and cyclic genes and cease the formation of new somites. Mechanistically, the interaction of PSM cells with the RGD motif of FN is required for dynamic formation of lamellipodia allowing motility and cell-cell contact formation, as these processes fail when wild type PSM cells are seeded into a FN matrix derived from FN^RGE/RGE^ fibroblasts. Thus, α5β1-mediated adhesion to FN in the PSM regulates the dynamics of membrane protrusions and cell-to-cell communication essential for elongation and segmentation of the body axis.

## Introduction

The vertebrate body axis elongates from anterior to posterior coinciding with the segmentation of the paraxial mesoderm into somites, which will form ribs, vertebral column and trunk muscles. Each pair of somites is sequentially separated from the anterior part of the presomitic mesoderm (PSM), with a period of approximately two hours in mice. The PSM appears as a loose and apparently unorganized mesenchyme at the caudal end of the embryo on both sides of the neural tube. A molecular oscillator, which results from the coordinated signalling of Wnt, FGF and Notch pathways, orchestrates the rhythmic definition of the site where the PSM segments. These pathways induce the transcription of several cyclic genes, whose dynamic expression domains sweep from the posterior to the anterior PSM with a periodicity that matches somite formation [1], [2], [3], [4], [5]. The perfectly timed expression of oscillating genes requires synchronisation of mesodermal cells [6]. The mechanism underlying the maintenance of synchrony is not well known. Studies in zebrafish [7], [8] and mouse [9], [10] led to the hypothesis that the transcriptional oscillations are generated spontaneously in cells of the PSM, and that cell-to-cell communications, such as those mediated by Notch/Delta signalling, keep neighbouring cells coupled. Concomitant with somite formation the posterior axis of the embryo has to continuously grow backwards to generate new paraxial mesoderm. It has recently been reported that the posterior tissue elongation depends on a graded posterior-to-anterior increase of cell density and decreasing motility of PSM cells [11]. The motility gradient is possibly also required to ensure a dynamic formation of cell-cell contacts between posterior PSM cells, which in turn sustains the coordinated expression of oscillating genes.

Cell motility and formation of cell-cell contacts depend on the dynamic organization of the actin cytoskeleton, which in turn is regulated by integrin binding to extracellular matrix (ECM) proteins. Integrins represent a major class of cell adhesion receptors [12] that bind to almost all ECM proteins including collagens and fibronectin (FN). Loss-of-function studies in mouse [13], [14], [15], [16], [17], chicken [18], zebrafish [19], [20] and *Xenopus* [21] have shown that integrins and FN play important roles during somite formation. The major FN binding integrins are α5β1 and αv-containing integrins. Disruption of the α5 integrin gene in mice arrests somitogenesis after the formation of 10–12 somites [13], [22]. Loss of αv expression does not affect somitogenesis [14], while mice lacking both α5 and αv integrins completely lack paraxial mesoderm segmentation [14], indicating that αv integrins compensate the absence of α5 integrins during the first cycles of segmentation.

FN also plays an essential role for somitogenesis, as loss of FN expression impairs somite formation in mice [16], zebrafish [19], [20] and *Xenopus* [21]. FN is a large dimeric glycoprotein consisting of three different types of modules called type I, type II and type III. In tissues such as the PSM secreted FN is assembled in an integrin-dependent manner into cross-linked, insoluble fibrils [23], [24], which provide a scaffold for the subsequent assembly of other ECM proteins such as collagens and fibrillins [25], [26], [27]. Integrin-binding to FN regulates a variety of cellular processes including migration, spreading, survival and proliferation. The RGD motif in the 10^th^ type III module (FNIII_10_) serves as major binding site for α5β1 as well as αv-containing integrins [28], [29], [30], [31].

It has recently been reported that the substitution of the aspartate (D) in the RGD motif with a glutamate residue (E) in the FN gene of mice (FN^RGE/RGE^) leads to the same defects as loss of the α5 integrin gene; somitogenesis is arrested around the 13^th^ somite stage and FN-RGE fibrils are assembled in an αv integrin-dependent manner through αv integrin binding to a novel binding site in FN [15]. To extend the previous analysis of FN^RGE/RGE^ mice we analyzed the somitogenesis phenotype of FN^RGE/RGE^ embryos. We report that several oscillating genes display a decreased, irregular and asymmetrical expression pattern leading to an arrest of body axis elongation and somite formation at the end of gastrulation (about E9.0). At this stage of development the posterior PSM expresses high levels of α5 integrin, which binds the RGD motif of FN. This interaction promotes a contractile, spindle-like shape of PSM cells *ex vivo*. The consequences of the shape defects are discussed.

## Materials and Methods

### Ethics Statement

The mice used for this study were kept in the animal house of the Max Planck Institute of Biochemistry. The analysis of RGE mice was carried out in strict accordance with all German (e.g. German Animal Welfare Act) and EU (e.g. Directive 86/609/EEC) applicable laws and regulations concerning care and use of laboratory animals. The Max Planck Institute of Biochemistry has a license for breeding and housing of laboratory animals (No. 5.1-568 - rural districts office). This includes the generation of knockout mice by ES cells injection. For this kind of experiments no separate licence or an approval of an ethics committee is required in the District Upper of Bavaria. All animals used were bred for scientific purposes. The Max Planck Institute of Biochemistry is registered at NIH and has a PHS Approved Animal Welfare Assurance from the Office of Laboratory Animal Welfare: #A5132-01 (see: http://grants.nih.gov/grants/olaw/assurance/500index.htm?Country=GM#GridTop).

### Mouse Strain

The generation of the FN^RGE/RGE^ mutant mouse strain has been described [15]. Mice were genotyped by PCR using the forward primer 5′-CAAAGAAGACCCCAAGAGCA-3′ and reverse primer 5′-ACAAGCCCTGGCCTTTAGTT-3′ to amplify a 250 bp fragment of the *FN* wild type and a 350 bp fragment with the *LoxP* site of the *FN^RGE^* locus.

### Immunohistochemistry

For histology embryos were isolated after timed matings, either fixed in 4% phosphate-buffered paraformaldehyde (PFA) or in Carnoy (60% ethanol, 30% chloroform, 10% acetic acid), embedded in paraffin, and sectioned at a 7 µm thickness. Tissue sections were blocked with 3% bovine serum albumin (BSA; Sigma-Aldrich)/PBS, incubated with primary antibodies in a humidity chamber over night at 4°C, then with either fluorescently labeled or biotinylated secondary antibodies for 1 h at RT and finally mounted.

### Antibodies

The following primary antibodies were used for immunofluorescence: rabbit anti-phosphoSer 10-histone H3 (1∶100; Upstate); rabbit anti-FN (1∶100; Chemicon); rabbit anti-β-catenin (1∶500; Sigma-Aldrich); rabbit anti-cleaved caspase 3 (1∶100; Cell Signalling); Cy3- or FITC-conjugated secondary antibodies (1∶200; Sigma-Aldrich or Jackson Immuno Research Laboratories). For immunohistochemistry was used phalloidin conjugated with Alexa488 (1∶200; Molecular Probes); and β-catenin with a biotinylated anti-IgG (1∶200; Vector Laboratories) as secondary antibody. Immunostaining was visualized with ABC Elite kit (Vector Laboratories) and a solution of 3,3′-diaminobenzidine (Sigma-Aldrich) and hydrogen peroxide. Images were taken with a Leica DMIRE2 confocal microscope with a 100× or 63× NA 1.4 oil objective, or with a Zeiss Axioplan microscope equipped with an Axiocam camera.

### In Situ Hybridization Probes

The digoxigenin-labeled RNA probes were generated by transcription with either T7 or T3 RNA polymerase from linearized template cDNA plasmids cloned in pBS vectors. The αv integrin probe represents a 336 bp fragment spanning nucleotide 1,462 to 1,798, the α5 integrin probe represents a 378 bp fragment spanning nucleotide 1,977 to 2,355 and the *Hes7* probe represents a 429 bp fragment spanning nucleotide 84 to 513. They were made by PCR using a mouse cDNA from E9.5 embryos as template. The plasmids carrying cDNA fragments for in situ hybridization for Pax3 and Pax1 were provided by Dr. Peter Gruss (Max-Planck Institute for Biophysical Chemistry, Göttingen, Germany); for Wnt3a by Dr. Wolfgang Wurst (Technical University, Munich, Germany); for FGF8 by Dr. Gail Martin (University of California, San Francisco, USA); for Notch1 by Dr. Tom Gridley (The Jackson Laboratory, Bar Harbor, Maine, USA); for Uncx4.1, Lfng, Axin2, Brachyury and Tbx6 by Dr. Bernhard Herrmann (Max-Planck Institute for Molecular Genetics, Berlin, Germany); and for Epha4 by Dr. Rüdiger Klein (Max-Planck Institute for Neurobiology, Martinsried, Germany).

### Whole Mount In Situ Hybridization

Staged embryos were isolated, fixed overnight at 4°C in 4% paraformaldehyde in PBS, rinsed in PBT (PBS, 0.1% Tween 20), dehydrated in methanol and stored at −20°C. Whole mount in situ hybridization was performed as described in [32].

### Preparation of FN Matrices and Video Microscopy of Tail Bud-derived Cells

Wild type and FN^RGE/RGE^ cells [15] cultured in serum replacement medium (47∶47∶5∶1 ratio of DME/Aim-V Medium (Invitrogen)/RPM1640/nonessential amino acids) were seeded on 50 µg/ml LM111-coated 35 mm dishes (5 µg/cm^2^; Roche) at a density of 5×10^5^ cells and allowed to produce a FN matrix with the endogenously expressed FN during 4–5 days. The cells were extracted after washing (100 mM Na_2_HPO_4_, pH 9.6, 2 mM MgCl_2_, 2 mM EGTA) by incubation for 60 min in lysis buffer (8 mM Na_2_HPO_4_, pH 9.6, 1% NP-40) at 37°C, two washes with 300 mM KCl, 10 mM Na_2_HPO_4_, pH 7.5, and four with water. The cell-free, pre-assembled 3D FN matrices were used as substrate to culture tail bud-derived cells, which were derived from tail buds dissected from about 25 E9.5 wild type embryos and trypsinized for 5 min at 37°C to obtain a single cell suspension. Subsequently trypsin inhibitor (50 µg/ml in PBS; Roche) was added, cells were centrifuged, suspended in serum replacement medium and seeded (10^4^ cells) onto wild type or FN-RGE matrices.

Video microscopy of tail bud-derived cells were recorded at 37°C and 5% CO_2_ on a Zeiss Axiovert 200 M (Zeiss) equipped with 10x/0.3, 20x/0.4 and 40x/0.6 objectives, a motorized stage (Märzhäuser, Germany), an environment chamber (EMBL Precision Engineering, Germany) and a cooled CCD camera (Roper Scientific, Princeton, NJ). Image acquisition and microscope control were carried out with Meta-Morph software (Molecular Devices, Downington, PA).

### Statistics

Results are expressed as the means±standard error (s.d.). Mann–Whitney U-statistics were used for comparisons between different data sets. Asterisks indicate significant differences (*P<0.01).

## Results

### Expression of FN^RGE/RGE^ Arrests Somitogenesis

Mice homozygous for the FN-RGE mutation (FN^RGE/RGE^) die between embryonic day (E) 9.5 and E10.5 [15] due to severe cardiovascular defects (manuscript in preparation). As a consequence of the cardiac malformations, some E9.5 FN^RGE/RGE^ embryos were severely affected in their development (see Fig. S1). For this study, we used FN^RGE/RGE^ embryos at ages between E9.0 and E10.0 with a head size that was not smaller than 30% of wild type littermates.

**Figure 1:**
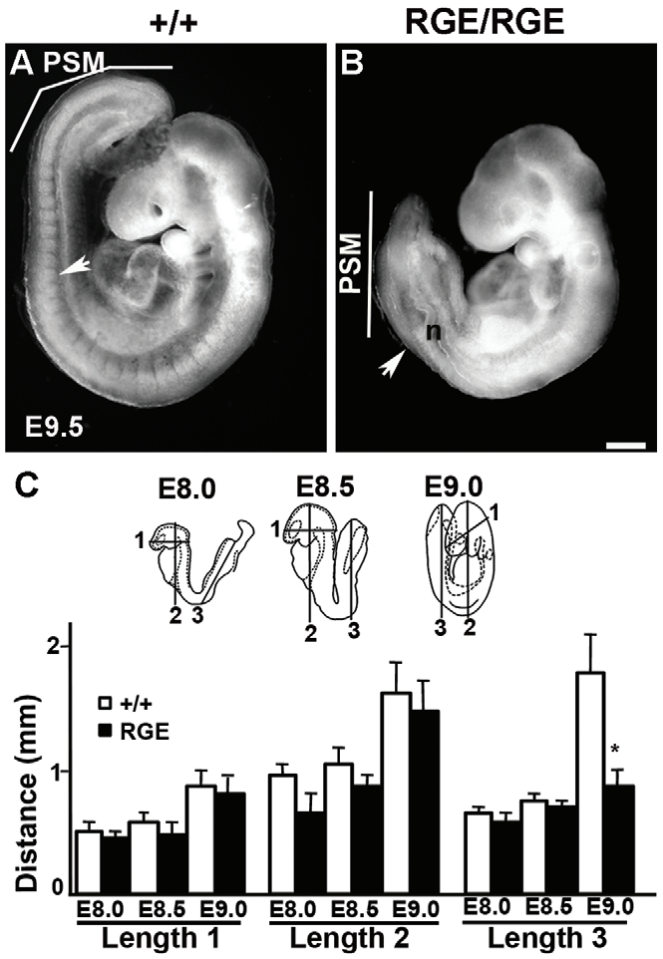
Defective axis elongation in FN^RGE/RGE^ embryos. (A,B) Whole-mount view of wild type and FN^RGE/RGE^ embryos at E9.5. The FN^RGE/RGE^ embryo displays a kinked neural tube (n) and a severely shortened and malformed posterior trunk. The arrow indicates the 13^th^ somite pair. A line marks the extension of the presomitic mesoderm (PSM). Note that the PSM layers in the FN^RGE/RGE^ embryo have irregular thickness. (C) Comparison of body dimensions between wild type (white bars) and FN^RGE/RGE^ (black bars) embryos at E8.0 (n = 7 for WT; n = 6 for FN^RGE/RGE^), E8.5 (n = 11 for WT; n = 9 for FN^RGE/RGE^) and E9.0 (n = 8 for WT; n = 6 for FN^RGE/RGE^). The cartoon indicates the areas used to measure tissue lengths: length 1, head; length 2, anterior half of the trunk; length 3, posterior half of the trunk. Numbers in parenthesis indicate the number of embryos analyzed. The scale bar represents 250 µm in (A,B).

The FN^RGE/RGE^ embryos display a shortened posterior trunk and an irregular PSM laterally expanded and malformed, thus impairing to complete the turning of the embryo. Their neural tubes are severely kinked, suggesting that the elongation rate of the neural tube was uncoupled from the paraxial mesoderm elongation rate (Fig. 1A, B). We measured FN^RGE/RGE^ embryos that had initiated turning; the length of the head and anterior trunk did not differ at E8.0, E8.5 and E9.0 from wild type littermates (Fig. 1C), but the posterior half of the trunk at E9.0 was reduced to about 47% of the wild type length. The decreased posterior trunk length is associated with a reduced number of somites (Table 1). At E8.0 and at E8.5 wild type and FN^RGE/RGE^ mice have developed about 4 and 7 somite pairs, respectively (Table 1). At E9.2 wild type embryos contained about 18 somites, while FN^RGE/RGE^ embryos had around 13 somite pairs. At E9.5 wild type embryos displayed around 21 somite pairs, while the FN^RGE/RGE^ embryos still contained around 13 somite pairs. We never detected FN^RGE/RGE^ embryos with more than 15 somite pairs. These findings indicate that the segmentation of the paraxial mesoderm into somites arrests at the end of Theiler stage 13 (E8.5–9.0), which corresponds to the period of embryo turning. Developmental defects became apparent during Theiler stages 14 and 15, and were most prominent in the developing heart.

**Table 1:** Number of somite pairs formed during development in wild type and FN^RGE/RGE^ embryos.

Approximated Embryonic Age	+/+	RGE/RGE
E8.0	4.0±1 (9)	4.0±1 (6)
E8.5	7.2±1 (10)	7.0±1 (10)
E9.2	18.2 ±2 (8)	13.5±1 (6)
E9.5	21.4±2 (20)	13.2±2 (15)

Number of somite pairs (mean±s.d.) formed in wild type and FN^RGE/RGE^ embryos between E8.0 and E9.5. Number of embryos analyzed is indicated in parenthesis.

### Anterior Somites Segregate and Differentiate in FN^RGE/RGE^ Embryos

**Figure 2:**
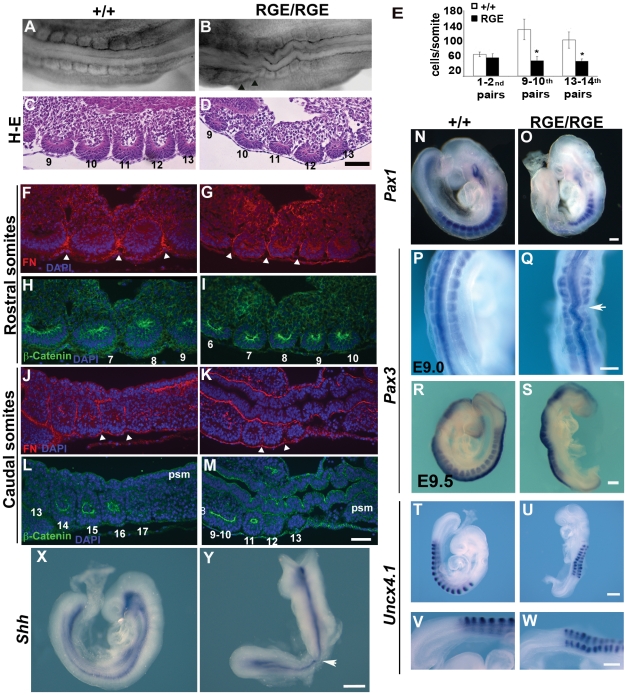
Anterior somites epithelialization and differentiation in FN^RGE/RGE^ embryos. Dorsal view of whole mount posterior trunk region of E9.5 wild type and FN^RGE/RGE^ embryos (A,B) and hematoxilin-eosin (H–E) staining of parasagittal paraffin sections corresponding to the pairs 9–13^th^ (C, D). The most posterior somites from FN^RGE/RGE^ embryo are smaller and asymmetrical in shape. (E) Quantification of cell numbers per section in somites 1^st^ and 2^nd^, 9^th^ and 10^th^, and 13^th^ and 14^th^ in E9.0 wild type (n = 21) and FN^RGE/RGE^ embryos (n = 18). (F–M) Immunofluorescence showing FN (red) and β-catenin (green) in the anterior or posterior somites from E9.2 wild type and FN^RGE/RGE^ embryos. Nuclei were stained with DAPI. In all panels the anterior side of the embryo is on the left. FN fibrils are deposited around the somites (arrowheads) but are less dense in FN^RGE/RGE^ embryos. β-catenin is expressed in the FN^RGE/RGE^ somites. (N–U) In situ hybridization of wild type and FN^RGE/RGE^ embryos with *Pax1* at E9.0 (n = 5; marks sclerotome; N,O), *Pax3* at E9.0 (n = 7; marks dermomyotome; P,Q) and at E9.5 (n = 3; R,S) and *Uncx4.1* at E9.2 (n = 5; marks the posterior parts of somites; T–W). All markers are expressed in the correct territories of FN^RGE/RGE^ somites. (X,Y) In situ hybridization of Sonic hedgehog expression at E9.0 (*Shh*; marks the axial mesoderm). The number of each somite pair is indicated in panels C, D, H, I, L and M. The scale bar represents 100 µm in (A–D), 50 µm in (F–M), 125 µm in (N–Q and T–Y) and 250 µm in (R,S).

**Figure 3:**
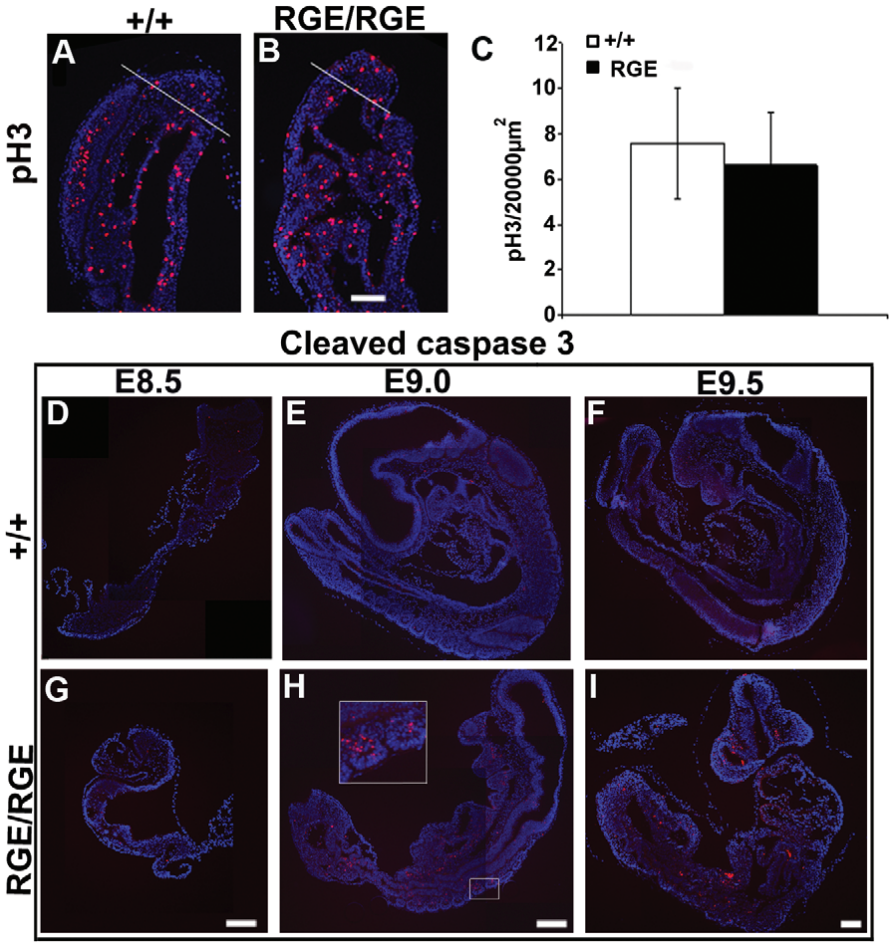
Proliferation and apoptosis in PSM of FN^RGE/RGE^ embryos. (A–C) Immunofluorescence for phosphorylated histone H3 (pH 3) in PSM of E9.0 wild type and FN^RGE/RGE^ embryos and quantification of pH 3-positive cells in the tail bud (n = 6 for WT and n = 9 for FN^RGE/RGE^). (D–I) Immunostaining for activated caspase 3 in E8.5, E9.0 and E9.5 wild type and FN^RGE/RGE^ embryos. Inset shows somites at higher magnification. The scale bars represent 100 *µ*m in (A,B) and 200 *µ*m in (D–I).

Next we tested whether the anterior somites in FN^RGE/RGE^ embryos are able to mature into epithelialized spheres with a central cavity, deposit a FN-rich ECM around their external boundaries and differentiate into defined territories. The first 8 somites in FN^RGE/RGE^ embryos had a normal size and morphology, while somites 9–13 were smaller than those of wild type littermates and had an asymmetrical size and shape (arrows in Fig. 2B). To determine whether the reduced size of somites 9–13 was associated with a diminished cell count, we determined the cell numbers in three consecutive sections of the 1^st^ and 2^nd^, 9^th^ and 10^th^, and 13^th^ and 14^th^ somites from E9.0 wild type and FN^RGE/RGE^ embryos. While cell numbers were unchanged in the 1^st^ and 2^nd^ somite pairs of the FN^RGE/RGE^ embryos, cell numbers of the 9^th^ and 10^th^ somite pairs were significantly reduced (Fig. 2E). These results indicate that the smaller size of the posterior somites in FN^RGE/RGE^ embryos was due to reduced cell content, which was likely due to the increased apoptosis rate (see below Fig. 3).

To determine whether the FN^RGE/RGE^ somites become epithelialized and segregated, we immunostained sections of anterior (5–10^th^) and the 5 posterior somite pairs from E9.2 embryos for FN and β-catenin expression (Fig. 2F–M). In both wild type and FN^RGE/RGE^ embryos the anterior as well as the posterior somites showed β-catenin expression in the apical cell border, indicating that somitic epithelialization proceeds normally in FN^RGE/RGE^ embryos (Fig. 2H, I, L, M). Furthermore, a FN matrix surrounded the somites, although the amount of FN appeared less dense in FN^RGE/RGE^ embryos (see arrow heads in Fig. 2G, K).

Following epithelialization somites commence their maturation and separate into a ventral mesenchymal part that differentiates into the sclerotome and expresses *Pax1*, and into a dorsal epithelial part that forms the dermomyotome and expresses *Pax3*. In situ hybridization with *Pax1* and *Pax3* probes revealed that at E9.0 and E9.5 wild type as well as FN^RGE/RGE^ embryos expressed *Pax1* in the ventral region (Fig. 2N, O) and *Pax3* in the dorsal region of their somites (Fig. 2P–S). The levels of *Pax1* and *Pax3* expression appeared less intense in some somites of FN^RGE/RGE^ embryos (see arrow in Fig. 2Q). The expression of *Uncx4.1* (Fig. 2T–W), which marks the posterior half of mature somites [33], [34] was similar between wild type and FN^RGE/RGE^ somites. *Pax1*, *Pax3* or *Uncx4.1* were neither expressed in the unsegmented PSM of E9.2 and E9.5 wild type nor of FN^RGE/RGE^ embryos (Fig. 2 and not shown). The expression of notochord-derived Shh [35], [36], [37], [38] was high along the notochord both in wild type and most FN^RGE/RGE^ embryos (Fig. 2X, Y). Some mutants displayed a lowered expression in distinct areas of the notochord (see arrow in Fig. 2X).

These findings indicate that the somites which formed in FN^RGE/RGE^ embryos are lined by a FN matrix, presomitic cells undergo a mesenchymal to epithelial transition and somites develop dorso-ventral as well as anterior-posterior polarities, suggesting that somite maturation can proceed in the absence of FN-α5β1 integrin interactions.

### Proliferation and Apoptosis in FN^RGE/RGE^ PSM

The arrest of axis elongation could be due to defects in proliferation or survival of PSM cells. To determine the number of proliferating cells in the PSM, we stained sections for the M phase marker pSer10-histone H3 (pH 3) [39]. We found similar numbers of pH 3-positive cells in somites and PSM of E9.0 wild type and FN^RGE/RGE^ embryos (Fig. 3A, B and data not shown). Quantification of pH 3-positive cells in the tail bud, the most posterior part of the embryo, revealed 7.76±2.40 pH 3-positive cells per 200 µm^2^ in the wild type versus 6.86±2.81 in FN^RGE/RGE^ embryos (Fig. 3C), indicating that FN-RGE does not significantly affect proliferation of PSM cells.

The rate of apoptosis was determined by counting activated caspase 3-positive cells. We found very few apoptotic cells in wild type embryos at E8.5, E9.0 and E9.5 (Fig. 3D–F). In FN^RGE/RGE^ embryos (Fig. 3G–I) the number of apoptotic cells was similarly low at E8.5 while small nests of apoptotic cells became visible in the neural tube and somites at E9.0 (see inset with two somites in Fig. 3H). At E9.5 the number of apoptotic cells further increased throughout FN^RGE/RGE^ embryos (Fig. 3I) and also became visible in the PSM. We assume that the continuous increase in apoptosis resulted from the vascular defects as the number of apoptotic cells increased with the severity of heart defects [15]. Therefore, apoptosis cannot explain the earlier arrest of axis elongation in FN^RGE/RGE^ embryos.

### Posterior PSM Cells Spread on FN in an α5β1-integrin-Dependent Manner

**Figure 4:**
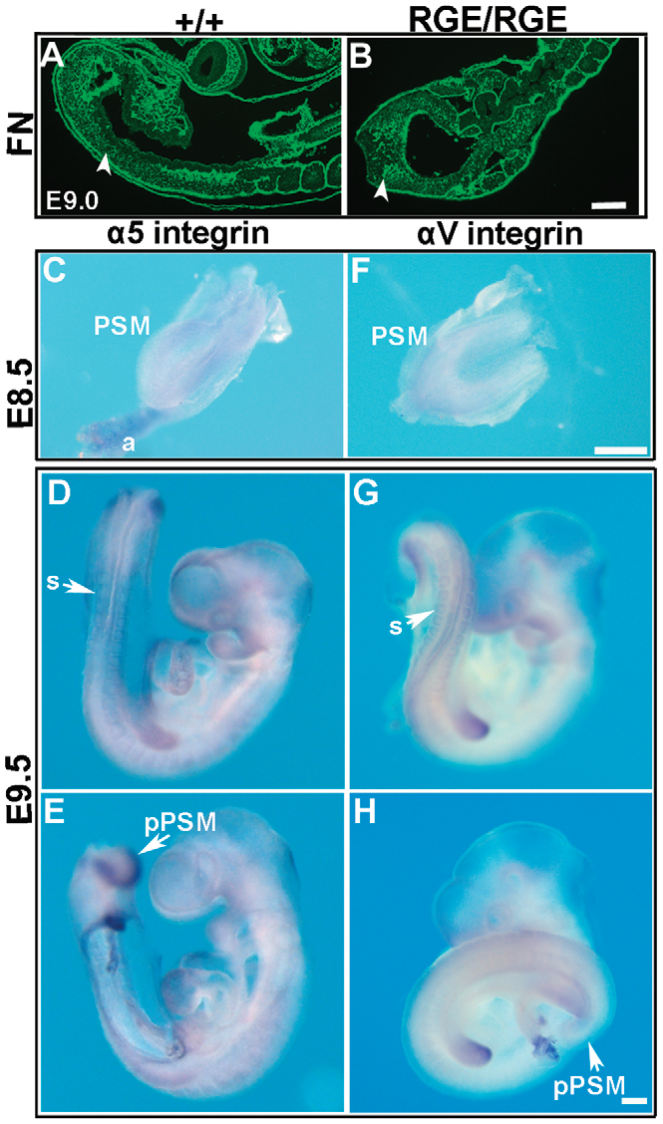
FN and α5 and αv integrins expression in the PSM. (A,B) Immunofluorescence for FN in E9.0 wild type and FN^RGE/RGE^ PSM. FN is abundantly deposited in the PSM of wild type and FN^RGE/RGE^ embryos (arrowheads). (C–H) In situ hybridization for α5 and αv integrins in E8.5 and E9.5 wild type embryos. At E8.5 both integrins are diffusely expressed in the PSM and in the allantois (a). At E9.5 α5 integrin is expressed around epithelial somites (s) and strongly in the posterior PSM (pPSM), while αv integrin is found around somites but not detectable in the posterior PSM (arrow) (for α5 n = 9 at E8.5 and n = 16 at E9.5; for αv n = 6 at E8.5 and n = 9 at E9.5). The scale bars represent 125 *µ*m in (A,B), and 250 *µ*m in (C–H).

FN fibrils were present around somite boundaries and in the PSM of control and FN^RGE/RGE^ embryos (Fig. 4A, B). Since FN fibril formation is mediated by integrins, we determined the expression of α5 and αv integrins in the PSM. At E8.5, we observed a weak and diffuse expression of α5 integrin in the PSM of wild type embryos (Fig. 4C). At E9.5, when axis elongation is mediated by the tail bud mesoderm, α5 integrin mRNA levels dramatically increased in the posterior PSM (Fig. 4D, E) and in the periphery of epithelialized somites, and remained low in the remaining embryo.

The expression of αv integrin was low and evenly distributed in the entire PSM of E8.5 wild type embryos (Fig. 4F). At E9.5 the αv integrin levels were high in limb buds, low around somites and not detectable in the PSM including the tail bud (Fig. 4G, H). The expression of both α5 and αv integrins was similar in FN^RGE/RGE^ embryos (not shown).

**Figure 5:**
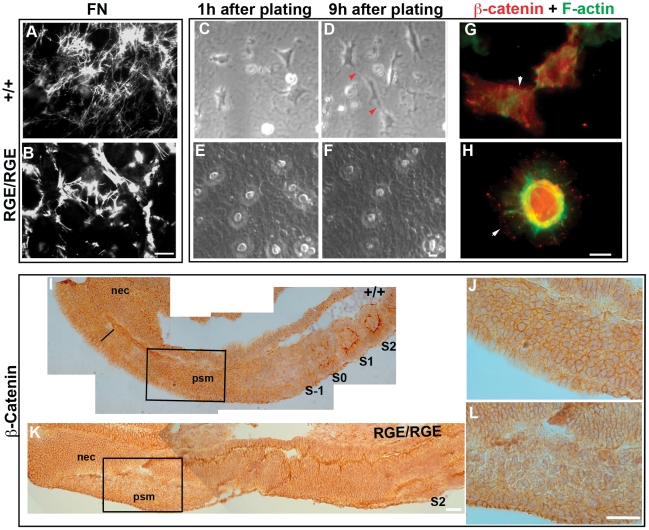
PSM cells spreading on FN-RGE matrices. (A,B) Immunofluorescence to FN of networks assembled by wild type and FN^RGE/RGE^ cells. Note the elaborated matrix produced by the controls with thin and long FN fibrils, whereas FN-RGE fibrils are thick and short. (C–F) Spreading of wild type tail bud-derived cells seeded into cell-free wild type (C,D) or FN-RGE (E,F) matrices. Images are snapshots from Videos S2 (C and D) and S3 (E and F) acquired at a rate of 1 frame per 5 min. The experiment was repeated 3 times. Note how cells adopt a fibroblast-like contractile shape on the wild type FN matrix making contacts with neighbouring cells (red arrows in D). The same cells on the FN-RGE matrix are flat, adopt a circular shape and make rare contacts with neighbouring cells. (G,H) Immunofluorescence of the F-actin cytoskeleton (green) and of β-catenin (red) of wild type tail bud-derived cells seeded into wild type (G) or FN-RGE (H) matrices. Note the presence of β-catenin at the cortex in cells seeded into the wild type FN matrix, and peri- and intranuclear staining in cells seeded into a FN-RGE matrix. (I–L) Immunostaining of β-catenin in PSM of E9.0 wild type and FN^RGE/RGE^ embryos. Low magnification image of wild type sections (I) show cytoplasmic staining of β-catenin along the PSM, width of which is shown by a line; the signal is uniform and intense in the posterior PSM, decreases in the anterior PSM (S-1) and shows an apical distribution in cells of somites (S0, S1…). The posterior PSM in FN^RGE/RGE^ embryo sections (K) shows less β-catenin at the cell cortex, while the adjacent neuroectoderm (nec) has normal β-catenin levels. Higher magnification (J and L) shows that cells in the posterior PSM of FN^RGE/RGE^ embryos have loose contact with neighbouring cells (L). The scale bars represent 15 *µ*m in (A–F), 5 *µ*m (G, H) and 50 *µ*m in (I–L).

The high expression of α5 integrin and FN in the posterior PSM prompted us to test whether the lack of a functional RGD motif in FN affects PSM cell behavior. As a first attempt we tried to compare posterior PSM cells motility and shape in vivo using time-lapse imaging of cultured embryos. Unfortunately, however, embryos did not survive culture in medium containing FN-depleted serum. FN depletion is a necessary prerequisite to avoid incorporation of plasma FN into FN^RGE^ fibrils of mutant tail buds. Therefore, we decided to analyze the behavior of wild type tail bud mesoderm-derived cells in a 3D FN matrix produced by either wild type or FN^RGE/RGE^ fibroblasts, respectively [15]. Wild type and FN^RGE/RGE^ fibroblasts were seeded on LN111 and allowed to secrete and assemble a wild type or FN-RGE matrix (Fig. 5A, B). Upon cell removal, freshly isolated tail bud cells from wild type E9.5 embryos were seeded into control or FN-RGE matrices and subsequently imaged by time-lapse microscopy (see Videos S1 and S2). Cells were cultured in serum replacement medium and no difference in their survival rate was observed between wild type and FN-RGE matrices during a period of 16 hours. Selected images from a representative movie (Fig. 5C–F) of tail bud cells in a wild type FN matrix show that the cells swiftly adopted a spindle shaped morphology with protruding and regressing lamellipodia that frequently contacted neighboring cells (Fig. 5D, arrowheads). Phalloidin staining showed actin patches at the plasma membrane and cables traversing the cytoplasm (Fig. 5G). In sharp contrast, the same wild type tail bud-derived mesoderm cells seeded into the mutant FN-RGE matrix adopted a flat circular shape with large lamellipodia around the entire cell that rarely regressed and rarely made contacts with other cells (Fig. 5E, F). The F-actin was enriched around the nucleus and extended thin cables towards the cell periphery (Fig. 5H). It has been reported that cell-cell contacts between mesenchymal cells in culture are produced by collisions of leading lamellae resulting in β-catenin-positive contact structures [40]. Indeed, immunofluorescence staining with specific antibodies showed β-catenin at the cell cortex and in connecting lamellipodia of neighboring mesoderm cells when seeded into a wild type 3D FN-matrix (arrow shows the cell cortex, Fig. 5G). Significantly, in cells seeded into a FN-RGE matrix β-catenin was mainly located in and around the nucleus (Fig. 5H)

To test whether contacts between FN^RGE/RGE^ PSM cells were also altered *in vivo* we analyzed the expression of β-catenin (Fig. 5I–L and not shown). At E9.0 wild type embryos showed strong β-catenin signal in epithelial somites restricted to the apical side of cells. Furthermore, we observed strong β-catenin expression at the cell cortex in the tightly packed posterior PSM cells. No detectable β-catenin staining was found in the anterior part of the PSM (Fig. 5I, J). In contrast, although the posterior PSM of FN^RGE/RGE^ embryos had β-catenin at the membrane, it was much less intense than in control embryos (Fig. 5K,L). Furthermore, the cells were less tightly attached to each other.

Altogether these results strongly support that the interaction of posterior PSM cells with FN by α5β1 integrins promotes spindle-like cell shape formation with dynamic lamellipodia protrusions and tight interactions with neighboring cells.

### Asymmetrical Expression of the “Segmentation Clock” and Cyclic Genes in FN^RGE/RGE^ Embryos

The formation of each new pair of somites from the anterior part of the PSM depends on morphogen gradients and the activity of signaling pathways that coordinate the cyclic expression of downstream genes in the PSM. Since integrin-mediated adhesion regulates a variety of signaling pathways, we next examined whether FN-RGE interferes with the activities of Wnt3a, FGF or Notch.

**Figure 6:**
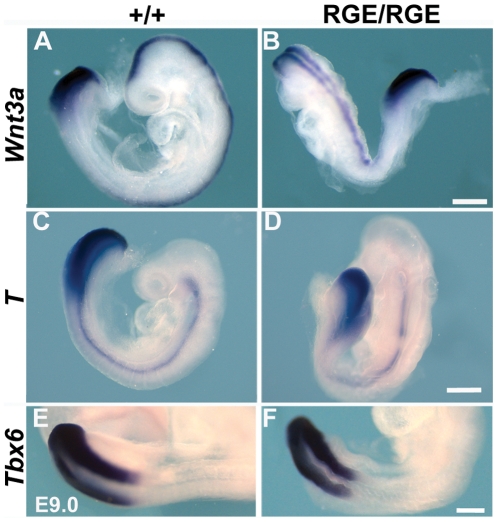
Wnt3a signalling in the PSM of FN^RGE/RGE^ embryos. In situ hybridization of *Wnt3a* expression (n = 6) (A,B) and downstream targets *Brachyury* (*T*; n = 5) (C,D) and *Tbx6* (n = 5) (E,F) in E9.0 wild type and FN^RGE/RGE^ embryos. The scale bars represent 250 *µ*m.

Wild type embryos expressed *Wnt3a* in the PSM in a posterior-to-anterior gradient and in the neural tube (Fig. 6A). The expression of *Wnt3a* was similar in FN^RGE/RGE^ embryos at E9.0, as was the expression of *Wnt3a* target genes such as *Brachyury* (*T*) and *Tbx6* (Fig. 6A–F). *Brachyury* was strongly expressed in notochord and PSM of wild type and most FN^RGE/RGE^ embryos (Fig. 6C, D). In a few FN^RGE/RGE^ embryos *Brachyury* expression appeared discontinuous in the notochord (arrow in Fig. 6D), which is likely due to the apoptosis observed in this tissue (Fig. 3H). These results exclude abnormal Wnt3a activity as cause for the somitogenesis defect.

**Figure 7:**
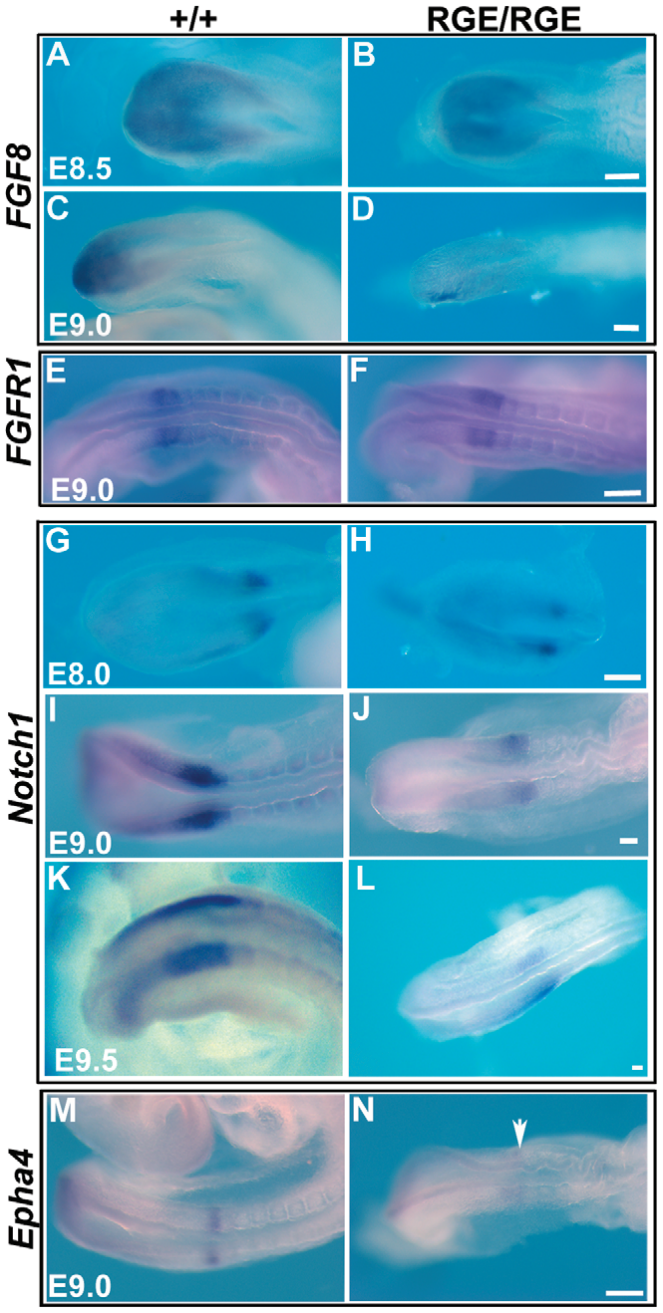
FGF8 and Notch1 pathways in the PSM of FN^RGE/RGE^ embryos. (A–F) Whole-mount in situ hybridization to show *FGF8* and its receptor *FGFR1* expression in E8.5 and E9.0 wild type and FN^RGE/RGE^ embryos (n = 5 for *FGF8* at E8.5; n = 8 at E9.0; n = 7 for *FGFR1*). (G–L) In situ hybridization of *Notch1* in E8.0, E9.0, and E9.5 wild type and FN^RGE/RGE^ embryos (n = 6 for wild type, and n = 8 for FN^RGE/RGE^ at E8.0, E9.0, and E9.5). (M,N) In situ hybridization of *Epha4* in E9.0 wild type and FN^RGE/RGE^ embryos (n = 4 for wild type and FN^RGE/RGE^). The scale bars represent 50 *µ*m.

The gradient of *FGF8* in wild type PSM differs significantly between E8.5 and E9.0 (Fig. 7A–D). At E8.5, *FGF8* is distributed in a medial-to-lateral gradient (Fig. 7A), while at E9.0 *FGF8* forms a posterior-to-anterior gradient and a marked expression in the tail bud (Fig. 7C). The FN^RGE/RGE^ embryos also displayed a similar medial-to-lateral gradient at E8.5 (Fig. 7B), while at E9.0 *FGF8* expression was bilaterally asymmetric and sometimes reduced (Fig. 7D and Fig. S2). The expression of *FGF4* and *FGFR1* in the PSM, however, was similar both at E8.5 (not shown) and E9.0 between wild type and FN^RGE/RGE^ embryos (Fig. 7E–F). These results indicate that α5β1 integrin interactions with the RGD motif of FN contribute to the control of FGF8 expression.

Wild type PSM showed a posterior-to-anterior gradient of *Notch1* expression with a stripe of *Notch1* mRNA in the most anterior PSM (Fig. 7G, I, K) marking the territory of the presumptive next somite [41]. In FN^RGE/RGE^ embryos *Notch1* expression was reduced and bilaterally asymmetric at E9.0 and E9.5 in the PSM and the presumptive somite area (Fig. 7J, L and Fig. S3). *Epha4* is a target gene of Notch/Deltall1 signaling expressed in the posterior PSM and marks the determination front, as Epha4 is expressed in the anterior border of the prospective somite [42]. E9.0 wild type embryos expressed *Epha4* in the posterior PSM and in a well-defined stripe marking the anterior half of the presumptive somite (Fig. 7M). In FN^RGE/RGE^ embryos the posterior PSM expression was present but the band in the presumptive somite was either absent or appeared diffuse (Fig. 7N). These findings indicate that Notch expression is regulated by α5β1 integrin binding to the RGD motif of FN and that the presumptive somite borders are ill-defined and diffuse in FN^RGE/RGE^ embryos.

**Figure 8:**
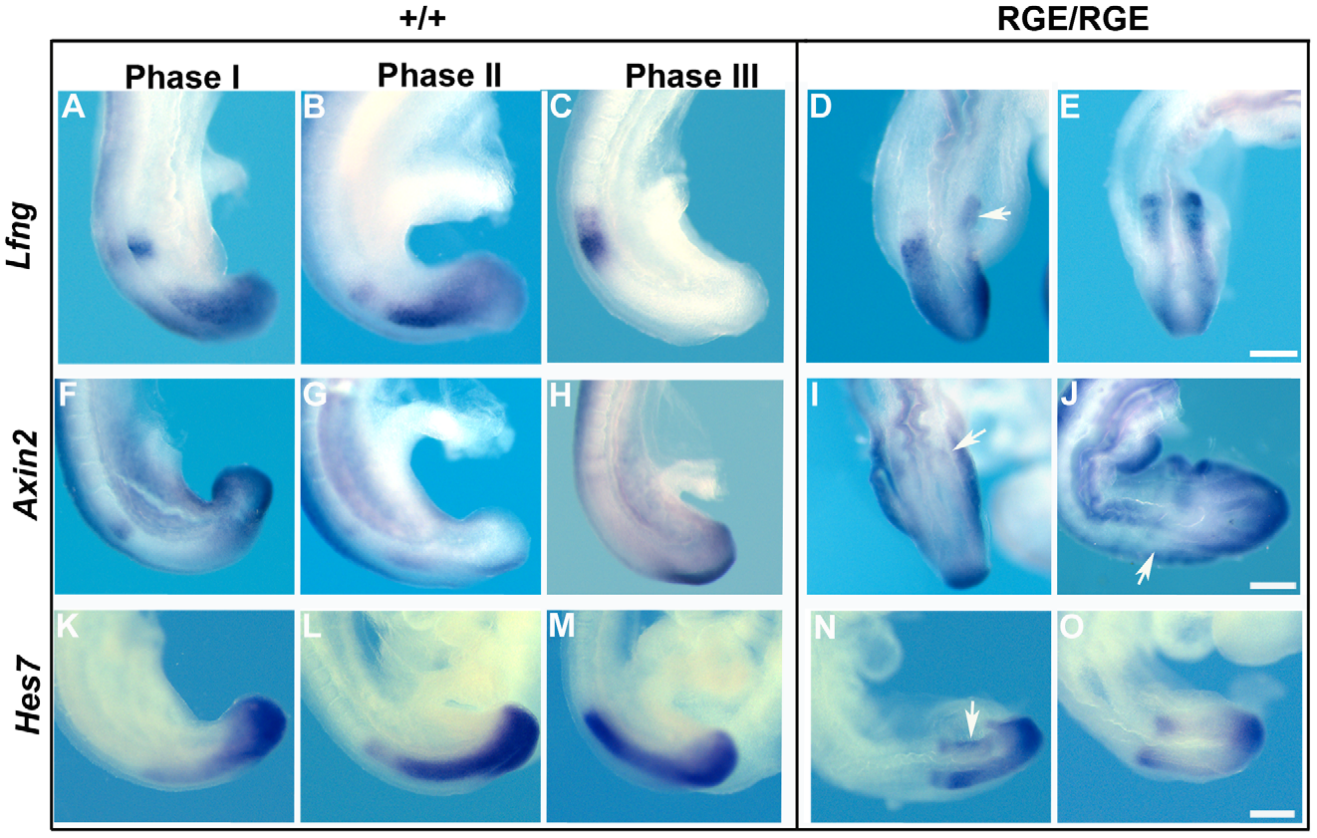
Expression of cyclic genes in the PSM of FN^RGE/RGE^ embryos. In situ hybridization of *Lfng* at E9.2 (A–E), *Axin2* at E9.5 (F–J) and *Hes7* at E9.2 (K–O) in wild type and FN^RGE/RGE^ embryos. In wild type embryos the three phases of the cyclic expression pattern along the PSM are shown (n = 27 for *Lfng*; n = 25 for *Axin2*; and n = 16 for *Hes7*). Note that the malformed posterior trunk of the FN^RGE/RGE^ embryos (D,E,I,J,N and O) allows dorsal views showing both layers of PSM, whereas lateral views are shown of wild type embryos (A–C, F–H and K–M). The *Lfng* expression in the FN^RGE/RGE^ embryos (D,E) is asymmetric and irregular displaying a typical salt-and-pepper expression pattern. *Axin2* and *Hes7* in FN^RGE/RGE^ embryos show abundant asymmetries (marked by arrows in I, J and N) (n = 13 for *Lfng*; n = 20 for *Axin2*; n = 9 for *Hes7*). The scale bars represent 250 *µ*m.

Next we determined the expression patterns of *Lfng*, *Axin2* and *Hes7*, representative cyclic genes regulated by the three signaling pathways. At E8.5 *Lfng* displayed a cyclic expression pattern that was similar in wild type and FN^RGE/RGE^ embryos (not shown). At E9.0–E9.5, however, the expression of *Lfng* cycled but was often bilaterally asymmetric in FN^RGE/RGE^ embryos (Fig. 8A–E), as the signals of *Lfng* were either in different phases of their expression cycle on the two sides of the PSM or low on one side. In several embryos we also observed an irregular, salt and pepper-like expression pattern of *Lfng* (Fig. 8D, E and Fig. S4). *Axin2* cycles out of phase with *Lnfg* and is both a target and a regulator of Wnt3a activity [43]. We readily detected oscillating expression of *Axin2* in E9.5 wild type embryos (Fig. 8F–J and Fig. S5). FN^RGE/RGE^ embryos also displayed oscillating *Axin2* expression, as we found embryos with all phases. The expression, however, always showed different intensities on the two sides of the mutant embryo (see arrows in Fig. 8I, J and Fig. S5). *Hes7* is downstream of FGF8 and Notch signaling [44] and its expression in E9.2 FN^RGE/RGE^ embryos was decreased and expression domains were irregular or asymmetric on both sides (see arrow in Fig. 8N). Retinoic acid signaling mutants also exhibit asymmetric expression patterns of cyclic genes, but always affecting to the same side. We discarded, therefore, a contribution of retinoic acid. Thus, in the absence of FN-α5β1 integrin adhesion expression of cyclic genes still oscillates but PSM cells have lost their synchrony.

## Discussion

The RGD motif of FN serves as major binding site for α5β1 and αvβ3 integrins [45]. Its functional ablation leads to cardiovascular abnormalities and arrest in axis elongation as well as somitogenesis. The defects resemble the phenotype of mice lacking α5β1 integrin expression.

The FN^RGE/RGE^ embryos show two remarkable features. First, they can develop the first 11–15 somites. Second, most genes of the somitic clock are expressed, albeit at reduced levels and with a bilateral asymmetry that affects both intensity and the oscillating phase. *Lfng* is most affected, but other genes such as *Hes7*, *Axin2*, FGF8 and *Notch1* also show asymmetric and decreased expression patterns. Only expression of Wnt3a and its downstream non-oscillatory targets is apparently normal.

Several papers reported defects in somite epithelialization in α5 integrin defective zebrafish [19], [20], [46]. In contrast, the somites in our FN^RGE/RGE^ embryos are well separated, undergo epithelialization and cavitation, and accomplish further steps of maturation, including dorso-ventral differentiation as shown by normal *Pax3* and *Pax1* expression as well as anterior-to-posterior polarity as shown by normal *Uncx4.1* expression. The anterior somites show no signs of deficient epithelialization, dissolving intersomitic boundaries or ectopic expression of differentiation markers (*Uncx4.1* or *Pax3* and *Pax1*) in the unsegmented PSM. The deposition of a continuous FN matrix that surrounds and separates the mutant somites is likely the reason for their normal formation and maintenance. FN fibrillogenesis is accomplished by α5β1 and αv-containing integrins [45]. They are both expressed in somitic cells. The ability of αvβ3 to assemble FN fibrils through RGD-dependent as well as -independent mechanisms [15] explains the presence of the FN matrix around FN^RGE/RGE^ somites and elsewhere in the mutant embryos. Why this does not occur in zebrafish is unclear.

An intriguing question is why both FN^RGE/RGE^ and α5 integrin deficient mutants [13] arrest somitogenesis after the formation of 12–15 somites. An explanation for the ‘late’ onset could be that α5 integrins do not play an important role for the extensive convergence movements that govern the early stages of PSM formation and gastrulation [47], while at later stages loss of α5 integrins leads to uncoordinated expression of somitic clock genes in the FN^RGE/RGE^ PSM blocking further somite segregation. Indeed, α5 integrin expression is weak in the early PSM and streak tissue (around E8.5). After gastrulation the PSM converts into a growth zone where mesoderm cells are continuously generated [3]. The expression of α5 integrin dramatically increases in the posterior PSM when the switch to this stage is occurring.

A remarkable feature of E9.0 or older FN^RGE/RGE^ embryos is the abnormal form of their short irregularly shaped posterior trunks and PSMs (Fig. 1, Fig. S4 and Fig. S5). It is possible that the lateral expansion occurs at the expense of its elongation or as consequence of an impaired elongation. The arrest in axis elongation seems to affect the paraxial mesoderm and not the neuroectoderm, as suggested by the kinked neural tube in FN^RGE/RGE^ embryos. We excluded proliferation defects as cause for this abnormality, as the numbers of phospho-histone as well as Ki67-positive cells were normal in the FN^RGE/RGE^ PSM at all stages analyzed. Furthermore, survival defects of PSM cells cannot account for the early defective elongation of the body axis. At E9.0 wild type as well as FN^RGE/RGE^ embryos displayed no significant apoptosis, while at E9.5 apoptotic cells became apparent throughout the FN^RGE/RGE^ embryos, which is likely due to nutritional deficit caused by the cardiovascular defect.

It has recently been shown that after gastrulation a cell density gradient is established in an anterior-to-posterior direction along the PSM, which is essential for axis elongation together with the PSM ECM extension [11]. Cell density decreases gradually allowing cells to acquire more motility, which did not consist of long and directional migrations as observed during gastrulation, but rather on short movements due to random protrusion and regression of lamellipodia in all directions [11]. The FN^RGE/RGE^ PSM lost the anterior-to-posterior cell density gradient. As a result, the PSM extension occurs in all directions resulting in a ball-like PSM that extends into all sides rather than forming a caudal bud structure. We made several findings that point to an important role of α5β1 integrin-mediated interaction with the RGD motif of FN for promoting or even maintaining the posterior PSM cell lamellipodia motility. First, the extent of motility in posterior PSM cells correlates with the expression levels of α5 integrin. Second, the PSM contains an intricate network of FN fibrils. Third, FN^RGE/RGE^ fibrils abrogate motility of PSM cells in vitro. In this experiment we seeded wild type, tail bud-derived cells into wild type or FN-RGE matrices. FN-RGE fibrils permit adhesion, which is mediated by αv-containing integrins [15], but fail to induce signals required for contractile, spindle-like cell phenotype, as seen on wild type FN fibrils. These findings are in agreement with reports demonstrating that fibroblasts lacking α5β1 induce high Rac1 activity and form lamellipodia but fail to activate RhoA at later stages which is required for adopting a contractile, motile morphology [48], [49]. Similar defects are observed with cells on FN-RGE fibrils suggesting that they fail to activate RhoA in the mutant ECM, which leads to impaired spreading and motility, and finally to a mainly lateral instead of a posterior expansion of PSM. Interestingly, FGF8 was shown to play an important role in regulating the cell motility gradient [11]. We observed diminished *FGF8* expression in the PSM, which is likely contributing to the motility defect. At current we do not know how FN-integrin signaling is controlling *FGF8* expression in the PSM. Wnt3a has been suggested to regulate *FGF8* expression, as mouse mutants lacking or expressing a stabilized version of β-catenin [50], [51] show opposing alterations in FGF8 gradient formation. The apparently normal *Wnt3a* levels in FN^RGE/RGE^ embryos exclude Wnt3a as a cause of the decreased and irregular *FGF8*. However reduced *Axin2* and *FGF8* expression has also been found in *Dll1*-null mice despite their normal *Wnt3a* and *T* expression [43]. Impaired Notch1-Deltall1 signaling in FN^RGE/RGE^ embryos could, therefore, be responsible for the *FGF8* decrease. However, the cell motility defect cannot be ascribed exclusively to FGF8 downregulation because defects in the dynamic of membrane protrusion are observed in wild type mesenchymal cells immediately after seeding on FN-RGE matrices. Of interest is also a recent report demosntrating that FN and α5 integrin are required for the morphogenesis and function of the node [52], which controls canonical left-right asymmetry in the mouse [53]. Although we did not investigate whether the development of the node is affected in FN^RGE/RGE^ embryos, we assume that potential defects in node morphogenesis and/or function will help explaining the heart malformations, but not the abnormal somite shape and the PSM genes expression asymmetries observed in E9.0 FN^RGE/RGE^ embryos.

In addition to the severe elongation defect of the posterior PSM, the anterior PSM fails to segregate into somites. We observed downregulation of Notch1 and decreased and/or asymmetrical expression of downstream cyclic genes such Hes7, Axin2 and Lfng. Juxtaposition of Epha4 and ephrin-B2-expressing cells is one of the requirements for boundary formation in the presumptive somite [54] and *Epha4* expression band was found to be weak and diffuse in FN^RGE/RGE^ embryos. In zebrafish Eph/Ephrin signaling controls α5β1 integrin clustering and thereby restricts FN assembly to somite boundaries [46]. Whether this is also the case in mouse is not clear. Moreover, the asymmetric phases of cyclic genes suggest that the synchronous induction of their expression is defective in the posterior PSM, probably before bilateralization, which is the site where α5 integrin expression is high. Several reports describe the postgastrulation posterior PSM as a static structure with few cell movements, which is believed to be important for ensuring synchrony and bilateral symmetry [6], [55], [56]. The necessity of a static structure seems contradictory with the need of graded cell motility for axis elongation. So why is the high motility important in the posterior part of the PSM? Interestingly, the expression of cyclic genes can be abrogated by treating the PSM with trypsin [4]. Trypsin treatment severely affects the integrity of the ECM resulting in abnormal cell-substrate adhesions, cell spreading and finally cell-cell communications. Similarly, loss of FN-α5β1 interaction in the PSM of FN^RGE/RGE^ embryos is also characterized by impaired cell membrane protrusions and cell-cell adhesion. The consequence of the impaired cell-cell contacts within the PSM of FN^RGE/RGE^ embryos may lead to impaired *Notch1* signaling and the induction of downstream cyclic genes, as Notch requires continuous on and off interactions with Delta on neighboring cells for maintaining robust signaling activity [10], [43], [44], [54], [57].

In summary, we report that α5β1 integrin-mediated interaction of PSM cells with the RGD motif of FN is essential to maintain dynamic cell lamellipodia formation, which in turn is required for cell-cell communications that permit axis elongation and the segmentation of the anterior PSM into somites.

## Supporting Information

Figure S1 **Heart malformation in E9.5 FN^RGE/RGE^ embryos.** Whole-mount view of wild type and FN^RGE/RGE^ embryos at E9.5. The FN^RGE/RGE^ embryo displays a severe heart defect leading to retarded growth. The scale bar is 250 *µ*m.
(TIF)

Figure S2 **FGF8 expression in FN^RGE/RGE^ embryos.** In situ hybridization of *FGF8* at E9.0 in a wild type and four FN^RGE/RGE^ PSMs. Note the variation of *FGF8* expression in FN^RGE/RGE^ embryos. The scale bar is 125 *µ*m.
(TIF)

Figure S3 ***Notch1*** **expression in E9.5 FN^RGE/RGE^ embryos.** In situ hybridization of *Notch1* in a wild type and five FN^RGE/RGE^ PSMs. Note the decreased or asymmetric expression pattern. The scale bar is 125 *µ*m.
(TIF)

Figure S4 **Lfng expression in E9.2 FN^RGE/RGE^ embryos.** In situ hybridization of *Lfng* in ten FN^RGE/RGE^ PSMs. Note the presence of cyclic *Lfng* expression but all mutant embryos show an asymmetric and heterogeneous, salt-and-pepper-like expression pattern. The scale bar represents 250 *µ*m.
(TIF)

Figure S5 **Axin2 expression in E9.5 wild type and FN^RGE/RGE^ embryos.** In situ hybridization of *Axin2* in three wild type and in nine FN^RGE/RGE^ PSMs at E9.5. The arrows indicate the position of the oscillant band. Note the presence of cyclic *Axin2* expression, but the mutant embryos show decreased and asymmetric expression pattern. The scale bar represents 250 *µ*m.
(TIF)

Video S1 Time-lapse recording at intervals of 5 min during 9 h of E9.5 wild type tail bud-derived cells seeded onto a wild type matrix. One representative experiment of three is shown.
(MPEG)

Video S2 Time-lapse recording at intervals of 5 min during 9 h of E9.5 wild type tail bud-derived cells seeded onto a FN-RGE matrix. One representative experiment of three is shown.
(MPEG)
