# Supplementary figures and images for: α5β1 Integrin-Mediated Adhesion to Fibronectin Is Required for Axis Elongation and Somitogenesis in Mice
Source: PLoS One. 2011 Jul 22;6(7):e22002. doi: 10.1371/journal.pone.0022002 (PMC3142108; doi:10.1371/journal.pone.0022002)

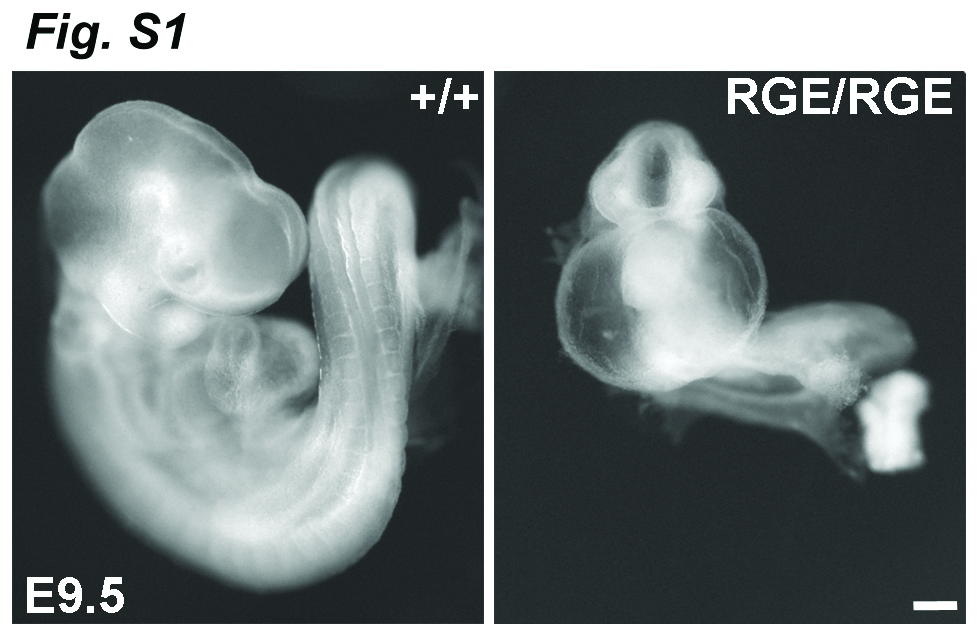

Supplement: Figure S1 — Heart malformation in E9.5 FNRGE/RGE embryos. Whole-mount view of wild type and FNRGE/RGE embryos at E9.5. The FNRGE/RGE embryo displays a severe heart defect leading to retarded growth. The scale bar is 250 µm. (TIF) [file pone.0022002.s001.tif]

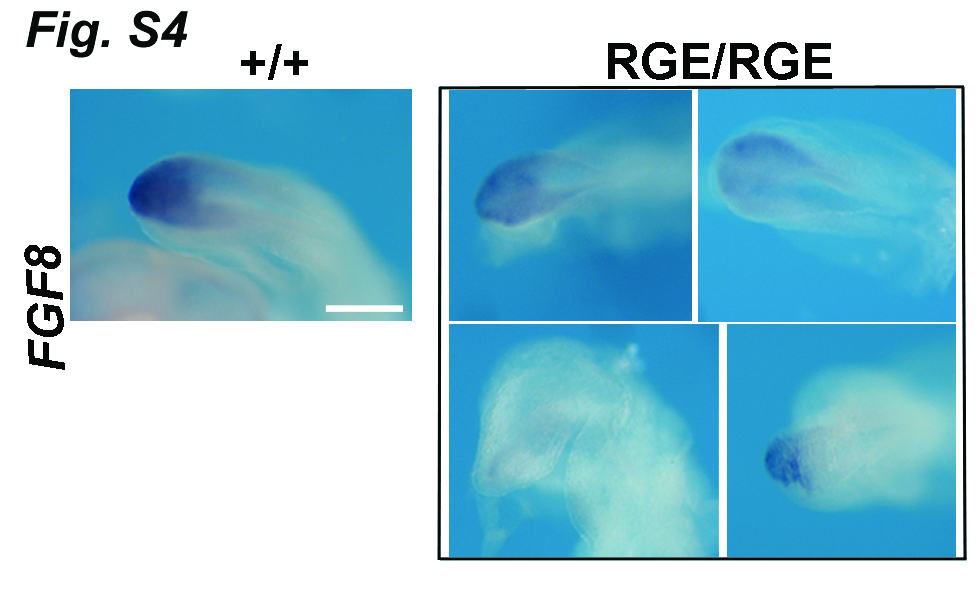

Supplement: Figure S2 — FGF8 expression in FNRGE/RGE embryos. In situ hybridization of FGF8 at E9.0 in a wild type and four FNRGE/RGE PSMs. Note the variation of FGF8 expression in FNRGE/RGE embryos. The scale bar is 125 µm. (TIF) [file pone.0022002.s002.tif]

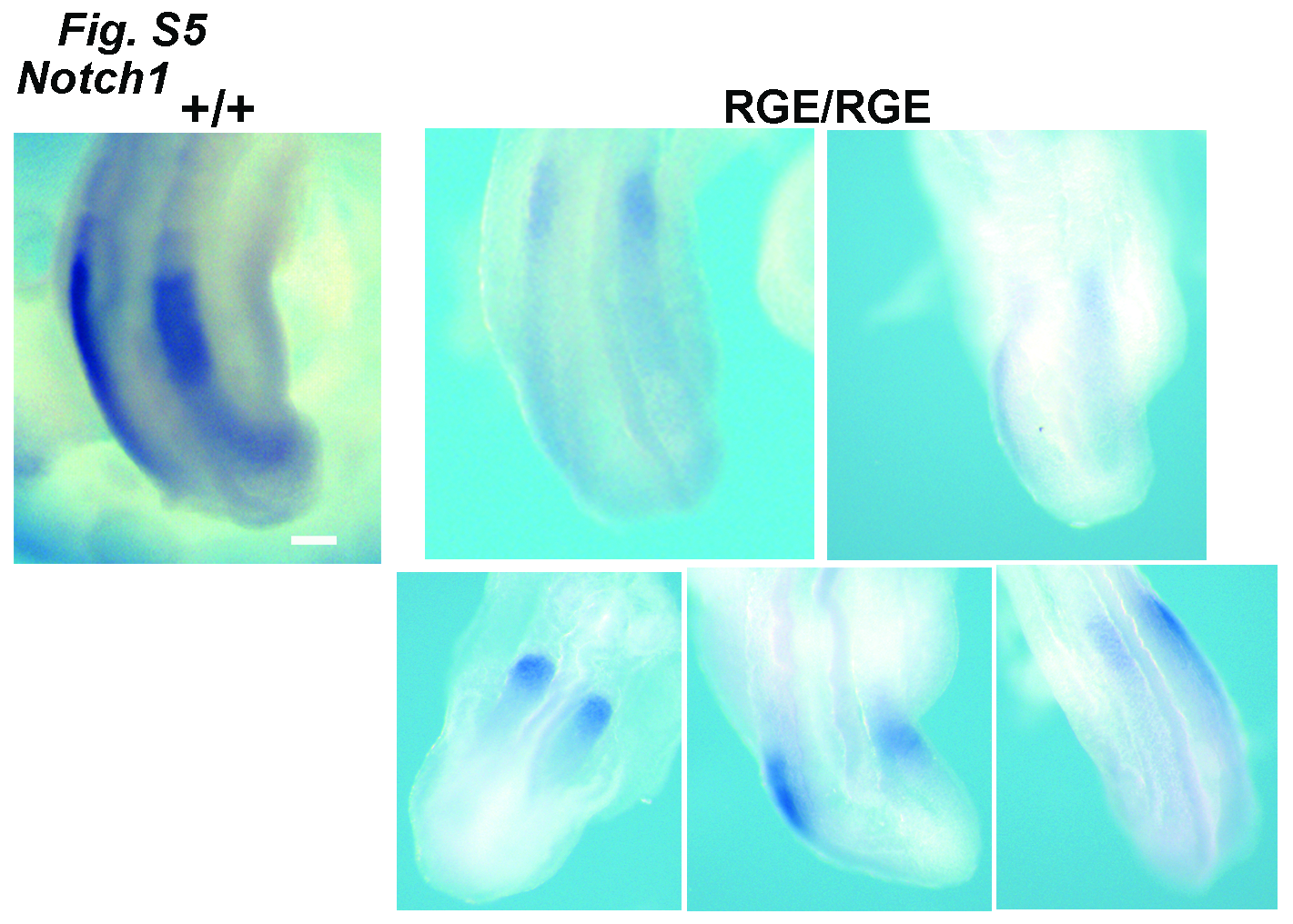

Supplement: Figure S3 — Notch1 expression in E9.5 FNRGE/RGE embryos. In situ hybridization of Notch1 in a wild type and five FNRGE/RGE PSMs. Note the decreased or asymmetric expression pattern. The scale bar is 125 µm. (TIF) [file pone.0022002.s003.tif]

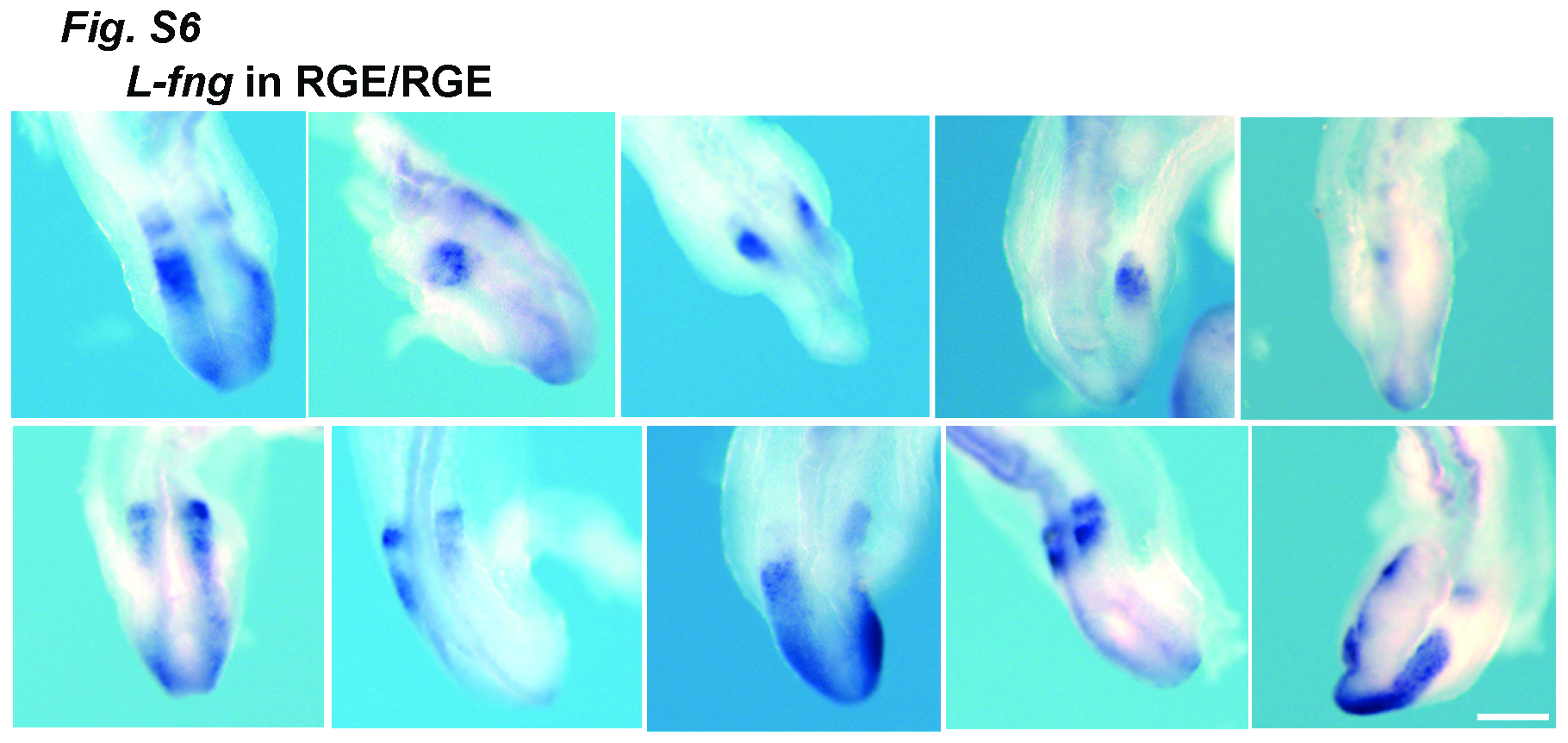

Supplement: Figure S4 — Lfng expression in E9.2 FNRGE/RGE embryos. In situ hybridization of Lfng in ten FNRGE/RGE PSMs. Note the presence of cyclic Lfng expression but all mutant embryos show an asymmetric and heterogeneous, salt-and-pepper-like expression pattern. The scale bar represents 250 µm. (TIF) [file pone.0022002.s004.tif]

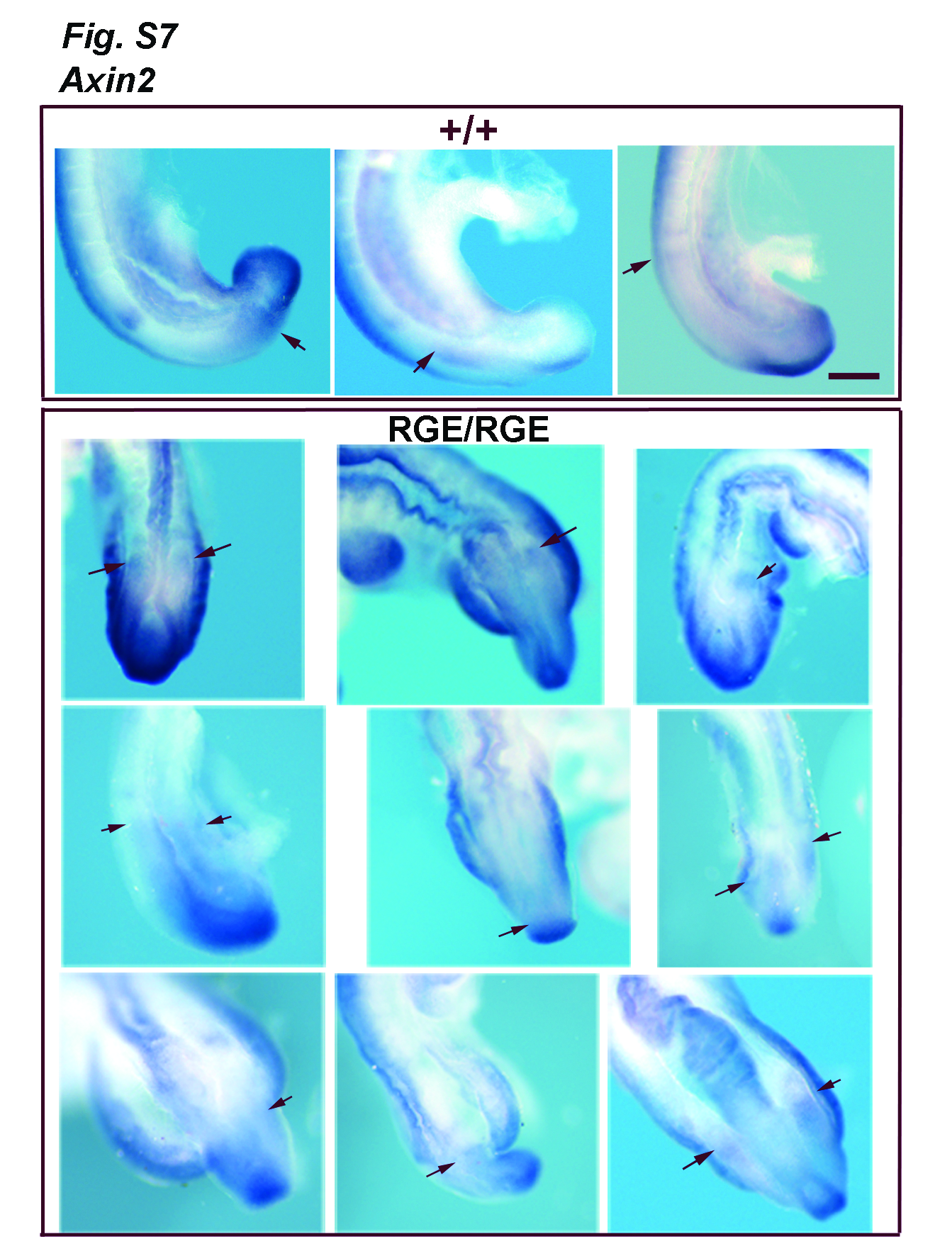

Supplement: Figure S5 — Axin2 expression in E9.5 wild type and FNRGE/RGE embryos. In situ hybridization of Axin2 in three wild type and in nine FNRGE/RGE PSMs at E9.5. The arrows indicate the position of the oscillant band. Note the presence of cyclic Axin2 expression, but the mutant embryos show decreased and asymmetric expression pattern. The scale bar represents 250 µm. (TIF) [file pone.0022002.s005.tif]
